# ACE2 as a Therapeutic Target for COVID-19; Its Role in Infectious Processes and Regulation by Modulators of the RAAS System

**DOI:** 10.3390/jcm9072096

**Published:** 2020-07-03

**Authors:** Veronique Michaud, Malavika Deodhar, Meghan Arwood, Sweilem B Al Rihani, Pamela Dow, Jacques Turgeon

**Affiliations:** 1 Tabula Rasa HealthCare Precision Pharmacotherapy Research & Development Institute, Orlando, FL 32827, USA; vmichaud@trhc.com (V.M.); mdeodhar@trhc.com (M.D.); marwood@trhc.com (M.A.); srihani@trhc.com (S.B.A.R.); pdow@trhc.com (P.D.); 2 Faculty of Pharmacy, Université de Montréal, Montreal, QC H3C 3J7, Canada

**Keywords:** ACE2, SARS-CoV-2, renin-angiotensin-aldosterone system, angiotensin II converting enzyme inhibitors, angiotensin II type 1 receptor blockers, pneumonia

## Abstract

Angiotensin converting enzyme 2 (ACE2) is the recognized host cell receptor responsible for mediating infection by severe acute respiratory syndrome coronavirus 2 (SARS-CoV-2). ACE2 bound to tissue facilitates infectivity of SARS-CoV-2; thus, one could argue that decreasing ACE2 tissue expression would be beneficial. However, ACE2 catalytic activity towards angiotensin I (Ang I) and II (Ang II) mitigates deleterious effects associated with activation of the renin-angiotensin-aldosterone system (RAAS) on several organs, including a pro-inflammatory status. At the tissue level, SARS-CoV-2 (a) binds to ACE2, leading to its internalization, and (b) favors ACE2 cleavage to form soluble ACE2: these actions result in decreased ACE2 tissue levels. Preserving tissue ACE2 activity while preventing ACE2 shredding is expected to circumvent unrestrained inflammatory response. Concerns have been raised around RAAS modulators and their effects on ACE2 expression or catalytic activity. Various cellular and animal models report conflicting results in various tissues. However, recent data from observational and meta-analysis studies in SARS-CoV-2-infected patients have concluded that RAAS modulators do not increase plasma ACE2 levels or susceptibility to infection and are not associated with more severe diseases. This review presents our current but evolving knowledge of the complex interplay between SARS-CoV-2 infection, ACE2 levels, modulators of RAAS activity and the effects of RAAS modulators on ACE2 expression.

## 1. Introduction

In late December 2019, several local health facilities in Wuhan City, Hubei Province of China, reported clusters of patients with pneumonia of an unknown etiology [1]. On 31 December 2019, the Chinese Center for Disease Control and Prevention (China CDC) dispatched a rapid response team to accompany Hubei provincial and Wuhan City health authorities; their mandate was to conduct an epidemiologic and etiologic investigation of the situation that appeared to be linked to a seafood and wet animal whole sale market [1]. In the first days of January 2020, Chinese scientists identified severe acute respiratory syndrome coronavirus 2 (SARS-CoV-2) as the responsible pathogen; a rapid sequencing of its RNA was also performed [2,3]. SARS-CoV-2 quickly spread in China and globally, causing the coronavirus disease 2019 (COVID-19) pandemic [4,5]. At the time this review was written (24 June 2020), COVID-19 has been diagnosed in more than 9,293,272 patients world-wide, has been associated with over 478,221 deaths, and has been considered a global health threat [6].

Soon after its outbreak, clinical features associated with COVID-19 started to be known. As of 2 January 2020, symptoms observed in 41 patients were fever (98%), cough (76%), myalgia and fatigue (44%) [7]. Similar observations were made following extraction of data from 1099 patients with laboratory-confirmed COVID-19 from 552 hospitals in 30 provinces in mainland China through 29 January 2020 [8]. The disease-causing capacity of this virus, along with its mortality rates—ranging from 0.1 to 15.4%—have raised concerns worldwide [1,6]. Clinical reports and epidemiological data related to COVID-19 indicate that older individuals with specific comorbidities have an increased risk of infection, potentially developing more severe symptoms, all of this resulting in higher mortality rates [9]. Hypertension, diabetes, and cardiovascular diseases seem to be the most frequent comorbid conditions in patients with COVID-19 [10,11]. Furthermore, concerns regarding the use of specific anti-hypertensive medications such as modulators of the renin angiotensin-aldosterone system (RAAS) and susceptibility to SARS-2-CoV infection have been raised and discussed [12,13,14,15,16,17,18]. Finally, a significant decrease in adherence to several drug classes—including RAAS modulators, antilipidemic and antidiabetic agents—has been observed since the beginning of the COVID-19 pandemic amidst an unrealistic optimism towards the pandemic, especially in men [19,20].

In this review, the interplay between SARS-CoV-2 infection, angiotensin converting enzyme 2 (ACE2) levels and modulators of the RAAS activity, as well as the effects of RAAS modulators on ACE2 expression, is addressed. A summary of the current knowledge as it pertains to the efficacy and safety of RAAS modulators in patients with all-cause pneumonia and underlying cardiovascular comorbidities is also presented.

## 2. Literature Search Strategy

We performed a systematic literature review from PubMed with significant keywords including COVID-19 and ACE2 (675 hits), COVID-19 and RAAS (52 hits), COVID-19 and ACE inhibitors (223 hits), COVID-19 and ARBs (71 hits), COVID-19 and mineralocorticoid inhibitors (6 hits), ACE2 and ACE inhibitors (1073 hits), ACE2 and ARBs (55 hits), ADAM17 and ACE2 (43 hits). From these 2197 publications, 220 references were retained and cited in the manuscript as major and primary sources of information. We also used our literature search daily to detect new publications and maintain our website as a source of information (https://trhc.spprdi.com/covid19/). Relevant and most recent publications were reviewed and information was extracted.

## 3. SARS-CoV-2

Coronaviruses, a part of the large *Coronaviridae* family, are large and enveloped viruses with single-stranded, positive-sense RNA genomes [21,22,23]. To date, seven coronaviruses have been identified and are known to cause diseases in humans (HCoVs) [24,25]. Coronaviruses are classified into four genera: Alpha, Beta, Gamma, and Delta [26]. HCoV-229E and HCoVNL63 belong to the Alphacoronavirus genus, while the Betacoronavirus genus includes HCoV-HKU1, HCoV-OC43, MERS-CoV (Middle Eastern Respiratory Syndrome), SARS-CoV, and the novel SARS-CoV-2. HCoV-NL63, HCoV-229E, HCoV-OC43, and HCoV-HKU1 are usually the cause of common colds, and in some cases cause severe lower respiratory tract infections [27]. Additionally, HCoV-NL63 infections are linked with croup (laryngotracheitis), while HCoV-OC43 infections are associated with severe lower respiratory tract infections in children [24,28,29]. The highly pathogenic SARS-CoV, MERS-CoV and SARS-CoV-2 are all zoonotic in origin, while the four low-pathogenicity coronaviruses (HCoV-NL63, HCoV-229E, HCoV-OC43, and HCoV-HKU1) are endemic in humans [30,31].

Initiation of viral infections involves the binding of a virus particle to host surface cellular receptors. Complete and comprehensive reviews of HCoV infectious processes have been reported [26,32,33]. In brief, for HCoVs, the process of activation (trigger for coronavirus to fuse membranes) and cellular entry is mediated by the surface-located spike (S) glycoprotein [26,34]. SARS-Co-V S protein is activated either by (1) lysosomal proteases (cathepsin L, cathepsin B) after endocytosis of the viral particle, or (2) extracellular proteases (e.g., elastases in the respiratory tract) for circulating viruses; or (3) by cell surface proteases (e.g., Type II transmembrane serine protease (TMPRSS2) on the surface of lung cells) [26,35,36,37,38,39,40,41,42]. The S protein comprises two functional subunits: subunit S1 binds to a receptor on the host cell surface for viral attachment, while subunit S2 fuses the host and viral membranes, allowing viral genomes to enter host cells [26,30].

**Figure 1:**
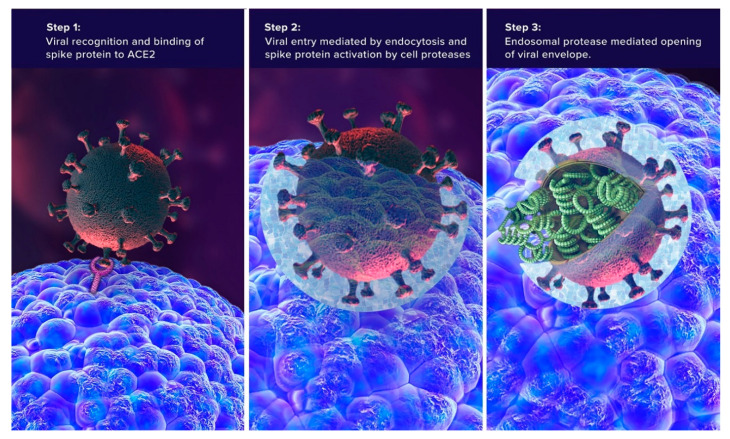
Interaction between ACE2 receptor and the SARS-CoV-2 virus (Contributed by Malavika Deodhar. Designed by Ernesto Lucio.)

Coronaviruses show different patterns of selective binding to host receptors. For example, SARS-CoV viruses (including SARS-CoV-2) specifically bind to the zinc-containing peptidase ACE2 (Figure 1) [43,44,45,46,47]. SARS-CoV binding does not interfere with the enzymatic activity of ACE2, nor does the enzymatic activity of ACE2 play any role in SARS-CoV entry [48]. The ACE2-virus complex is then translocated to endosomes where endosomal acid proteases cleave the S protein, activating its fusion and release of the viral genome [46,49,50,51,52]. Viral entry of SARS-CoV-2 via ACE2 receptors leads to pneumonia, acute myocardial injury, and chronic damage to the cardiovascular system [53,54]. Recently, nasal gene expression of ACE2 has been shown to be lower in children than in adults, which may explain age-related differences in the risk associated with SARS-CoV-2, at least for upper respiratory tract infections [55].

Though SARS-CoV and SARS-CoV-2 share a common mechanism for entry into the cell, SARS-CoV-2 differs from SARS-CoV by substitutions in 380 amino acids [56]. There are 14 critical amino acids for ACE2 binding in the receptor-binding domain (RBD) of SARS-CoV-2, of which 6 differ between SARS-CoV-1 and SARS-CoV-2 [30]. These alterations provide improved hydrophobic interactions and salt bridge formations, making the binding affinity between SARS-CoV-2 and ACE2 stronger than the original SARS-CoV. Stronger binding could be an underlying factor explaining the larger global impact of COVID-19 compared to the SARS pandemic in 2003 [57,58]. Blocking the binding of SARS-CoV-2 to human ACE2 by interfering with the RBD of the viral S-protein could be a potential therapeutic target [59].

## 4. The Renin-Angiotensin-Aldosterone System (RAAS)

**Figure 2:**
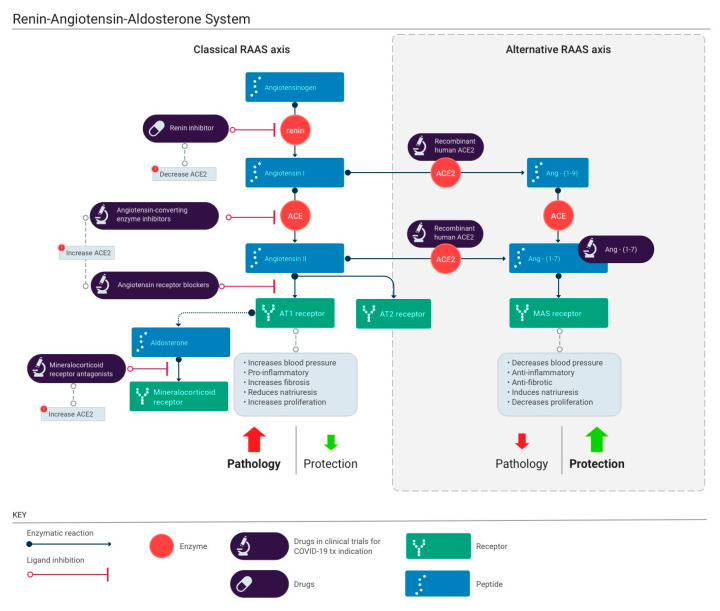
The renin-angiotensin-aldosterone system (RAAS) cascade, RAAS modulator actions and their potential impacts on ACE2 (Contributed by Malavika Deodhar, designed by Ernesto Lucio.)

Significant research initiatives have created a better understanding of both the complexity of the RAAS and the involvement of multiple enzymes and receptors in these pathways (Figure 2) [60,61]. Over the last century, we have learned that RAAS is stimulated by hypotension, ultimately resulting in the production of angiotensin II (Ang II or Ang-1–8) to increase blood pressure via multiple pathways. Renin is a proteolytic enzyme that cleaves angiotensinogen in plasma to angiotensin I (Ang I or Ang-1–10). Ang I further cleaves to Ang II via the angiotensin converting enzyme (ACE). The biologically active peptide, Ang II, acts on angiotensin type I and type II receptors (AT1R and AT2R). Binding of Ang II to AT1R promotes vasoconstriction, inflammation, renal sodium and water reabsorption and oxidative stress [62]. From the mid-1980s, research has recognized that a local autocrine/paracrine RAAS exists in a number of tissues which may play a significant role in regulating locally (tissue) vs. systemically (serum) the RAAS [63,64,65]. Collectively, these discoveries have stimulated the development of therapies targeting various proteins in the RAAS [66].

## 5. Role of ACE2 in the RAAS

ACE2 is an important constituent of the RAAS and, as mentioned previously, is essential for the entry of SARS-CoV-2 into cells [44,46,67,68,69]. Recent data obtained from COVID-19 patients has demonstrated a significant increase in Ang II levels, which was linearly and positively correlated with both viral load and lung injury [70]. With elevated levels, more Ang II is available to bind to the AT1R, thereby mediating increased vasoconstriction, inflammation, and lung vascular permeability [71,72]. 

Detrimental effects associated with the overstimulation of the Ang II/AT1R axis have spurred research for a counter-regulatory axis of the activated RAAS. ACE2 is a RAAS regulator able to mitigate the detrimental actions mediated by Ang II and AT1R activation [73]. ACE2, a membrane-bound carboxypeptidase, has a fundamentally protective role in regulating cardiovascular and renal functions [74]. ACE2 is a homolog of ACE, with the catalytic domains of both peptides sharing approximately 40% identity [74,75]. While ACE increases the production of Ang II, ACE2 reduces it. This is achieved via two mechanisms (Figure 2). First, ACE2 shuttles the conversion of Ang I to Ang-(1–9), instead of Ang II [73,74]. Second, ACE2 directly acts on Ang II and converts it to the vasodilator Ang-(1–7). As a result, ACE2 acts to increase production of Ang-(1–7) at the expense of Ang II. Ang II acts through AT1Rs and AT2Rs, while Ang-(1–7) mediates its effects via Mas receptors (MasR), exerting vasodilatory and anti-proliferative effects [68,76]. Other protective effects of the ACE2/Ang-(1–7)/MasR axis include reductions in the release of pro-inflammatory cytokines and both anti-fibrotic and anti-hypertrophic effects [77].

While ACE expression is ubiquitous in the body, ACE2 shows a more restricted distribution [78,79]. ACE2 is expressed specifically in the heart, kidneys, testes, gastrointestinal tract (esophagus, enterocytes of the small intestine and colon), arterial and venous endothelial cells, lungs, cholangiocytes, and bladder [75,80,81,82,83,84,85]. The tissue expression and distribution of ACE2 could help delineate the potential infection routes of SARS-CoV-2, as the main targets of SARS-CoV-2 are the lungs, immune organs, and systemic small vessels. At the same time, ACE2 distribution is large enough to explain the increased potential for multiple organ damage or failure as a consequence of SARS-CoV-2 infection [80]. 

In 2004 and 2005, Hamming et al. and Jia et al. studied the host–pathogen interactions of SARS-CoV and NL63 coronavirus and reported that ACE2 protein was expressed in human airway epithelia and in lung parenchyma [82,86]. It was shown that the ACE2 protein was more abundantly expressed on the apical side than the basolateral side of polarized airway epithelia, which could favor viral entry from respiratory droplets [86]. ACE2 expression in the lung was also confirmed in recent single cell RNA-seq analysis performed on lung tissue donated from eight healthy adults [87]. This study demonstrated that 83% of lung cells expressing ACE2 were alveolar epithelial type II cells. It also suggests that alveolar cells can serve as a reservoir for SARS-CoV-2 infection, corroborating previous evidence [82]. ACE2 receptors being found in cells in the lower lung can explain the high incidence of pneumonia and bronchitis in patients with severe SARS-CoV-2 infection, though its physiological role in the airway is currently unknown. 

High ACE2 RNA levels have also been identified in oral mucosa (especially in epithelial cells of the tongue) [88]. Case reports indicate sensory impairments in patients tested positive for SARS-CoV-2; loss of smell and loss of taste have anecdotally been observed in a significant number of patients [89]. A pilot study showed that rectal specimens have tested positive for SARS-CoV-2 (4 out of 62); the virus was also detected in the gastrointestinal tract, saliva, or urine [8,90]. Tissue expression of ACE2 may explain some extrapulmonary manifestations, such as gastrointestinal symptoms observed with HCoV infections like watery diarrhea [91,92].

Beside its expression in specific tissues, ACE2 is also observed in plasma. Proteolytic cleavage of the membrane bound ACE2 into the soluble form is in part dependent of the enzyme ADAM17 [93,94]. Cleavage of tissue ACE2 would result in a loss of ACE2 protection against tissue RAAS by decreasing the compensatory potential of ACE2. ADAM17 activity is thought to be higher in men than women [95]. Studies have shown that SARS-CoV viruses activate ADAM17, thus explaining increased plasma levels of ACE2 observed in patients with SARS and COVID-19 [96]. Activation of ADAM17 is damaging since, (1) it reduces the levels and cardioprotective effects of ACE2 in tissues, and (2) it triggers an uncontrolled inflammatory response [97]. As opposed to the tissue-bound ACE2, the role of the circulating soluble ACE2 is not fully known. The increase in circulating soluble ACE2 might represent a pathological shift of ACE2 from its “normal” tissue membrane location.

## 6. ACE2 Regulation

Evidence suggests that ACE2 is regulated by various pathways [98,99]. Though evidence for the coregulation of ACE and ACE2 levels is not robust, studies have suggested a close relationship between Ang II levels and ACE2 expression [100]. For instance, disease states involving dysregulation of the RAAS have been reported to play a role in regulating ACE2 levels [101,102]. Plasma levels of ACE2 are reported to be very low or undetectable in healthy subjects, but in the presence of cardiovascular diseases, a significant increase in ACE2 levels was observed [96,101]. Alterations in the RAAS axis are believed to be critical for the development of diabetic nephropathy (micro- and macro-vascular complications) [103,104]. For example, in diabetic renal tubules, *ACE2* gene expression is decreased by approximately 50%, which is associated with reduced Ang-(1–7) formation and Ang II accumulation [83].

Sex-related differences have been observed between men and women in mortality rates from SARS-CoV and SARS-CoV-2; the death rate is higher in men than in women [105,106]. This difference could be attributed to increased ACE2 expression in men, leading to ACE2 plasma levels that are higher in men than in women [96]. Differences between men and women could also be related to a genetic polymorphism in *ACE2.* Recessive *ACE2* polymorphisms predisposing men to infection could be silenced in women as *ACE2* is located on the X chromosome in the human genome (if only one X chromosome is affected) [67]. Indeed, several studies have reported that *ACE2* genetic polymorphisms can play a role in cardiovascular diseases, particularly in gender-related hypertension susceptibility [107,108]. *ACE2* polymorphism could also explain in part ethnic differences observed in COVID-19 disease prevalence and severity [109]. For instance, East Asian populations were found to have much higher allele frequencies in the expression quantitative trait locus variants associated with higher ACE2 expression in tissues than other populations; other variants also showed important inter-ethnic differences [110]. Finally, some genetic variants observed in *ACE2* leading to variations in the intermolecular interactions with the viral S-protein may confer resistance [109].

Other studies have suggested an involvement of aldosterone and estrogens in regulating ACE2 expression [99,111]. Evidence supports that estrogens can modify the local RAAS homeostasis via the downregulation of ACE and simultaneous upregulation of ACE2, AT2R, and MasR expression levels [107,112]. Finally, data from animal experiments conducted with infected SARS-CoV mice suggest that estrogen receptor signaling plays a pivotal role for protection in females [113].

## 7. Blocking or Enhancing ACE2?

ACE2 is an important regulator of the immune response, especially inflammation [114]. A 2014 study found that the ACE2 enzyme offers protection against lethal avian influenza [115]. In acute lung injury, ACE, Ang II, and the AT1R function as lung injury-promoting factors, while the negative regulation of Ang II levels by ACE2 protects against lung injury [71,116,117,118,119,120,121,122,123,124,125,126]. Paradoxically, ACE2 is identified as a functional receptor essential for SARS infections [114]. The burden of viral replication triggers the immune response through a downregulation of ACE2 expression by SARS-CoV S-protein, leading to pathogenesis of severe acute lung injury [72,127,128]. So, even though ACE2 facilitates SARS-CoV-2 entry in pulmonary epithelial cells, ACE2/Ang-(1–7) axis stimulation could mitigate SARS-induced pulmonary injuries and the severity of damage [114]. ACE2 internalization by SARS-CoV-2 could potentially result in the loss of ACE2 at the cell surface, canceling a major pathway for the cell to degrade Ang II and generate Ang-(1–7) [71,72,129]. Subsequently, ACE2 reduction due to its internalization may increase the Ang II/Ang-(1–7) ratio, which may worsen the pulmonary damage first triggered by the SARS-CoV-2 infection. At lung level, such dysregulation could ease the progression of inflammatory and hyper-coagulation responses that are reliant on local overactivity of Ang II, inefficiently counterbalanced by Ang-(1–7) [114]. For instance, in vivo data in SARS-CoV infected mice revealed exacerbation in acute lung failure by the downregulation of ACE2 expression [72]. In addition to lung injury seen in COVID-19 patients, SARS-CoV-2 has been shown to cause acute myocardial injury, the mechanism of which may be related to ACE2 since ACE2 is highly expressed in the heart [130]. 

The general cardioprotective role of ACE2 has limited the development of ACE2 inhibitors because they are unlikely to be of therapeutic benefit in cardiovascular disorders where the upregulation of ACE2 expression and activity is beneficial. Even so, a number of ACE2 selective inhibitors have been developed, including MLN-4760 (GL1001), DX-600, and 416F2 [131,132,133]. On the contrary, ACE2 activators, such as xanthenone, have been tested in a heart failure rat model [134]. Xanthenone has been demonstrated to decrease blood pressure and improve cardiac function in spontaneously hypertensive rats. However, the potential therapeutic effects of ACE2 activators or analogs needs to be further investigated since antibodies against ACE2 have been detected in human plasma and development of autoantibodies to ACE2 may be associated with detrimental effects such as constrictive vasculopathies [135].

## 8. ACE Inhibitors

In the mid-1970s, the search for a potent, orally active, ACE inhibitor led to the synthesis of captopril by researchers at E.R. Squibb & Sons Pharmaceuticals [136]. This was followed by the synthesis and introduction to the market of a series of other ACE inhibitors with various pharmacokinetic and pharmacodynamic properties [137].

In the mid-1980s, the concept of two classes of ACE systems—serum ACE and tissue-specific ACE—was introduced [63,64,65]. The presence of angiotensin and renin messenger RNA (mRNA) was demonstrated in 12 different extrahepatic tissues of rats, strongly suggesting that there is local synthesis of angiotensinogen and renin [138,139]. Similar to the systemic ACE system (involving angiotensinogen released from the liver, its serum conversion into Ang I by renin released from the kidneys and, through a passage into the pulmonary vasculature, the conversion of Ang I into Ang II), tissue RAAS is capable of both local generation and action of Ang II [140,141,142,143]. Numerous studies, including large clinical trials, have demonstrated the greater value of tissue ACE inhibition vs. serum ACE inhibition in patients with hypertension, diabetes, renal disease, and heart failure [144]. Drug biophysical characteristics were tentatively associated with increased affinity for ACE, as carboxyl-containing ACE inhibitors (enalapril, lisinopril, trandolapril, ramipril, quinapril, perindopril) were demonstrated to bind more strongly to the zinc ligand than sulphydryl-containing (captopril) or phosphoryl-containing (fosinopril) ACE inhibitors [145]. More importantly, ACE inhibitors lipophilicity was associated with greater selectivity and affinity for tissue ACE: the drug lipophilicity is ranked captopril < lisinopril < enalapril < perindopril < ramipril < quinapril < trandolapril < fosinopril [144]. 

## 9. Angiotensin Receptor Blockers (ARBs)

As mentioned previously, Ang II actions are mediated by binding mostly to two receptors, AT1R and AT2R [146]. These receptors are members of the G protein-coupled receptor family and interact with specific G-proteins, leading to the activation of special effector systems.

Saralasin (a partial agonist) and other Ang II peptide analogues were studied as potential ARBs [147,148,149]. Due to their poor bioavailability, molecular modeling strategies were used to derive orally active, potent, and selective nonpeptide ARBs. At least eight ARBs have been introduced into clinical practice: losartan, valsartan, azilsartan, candesartan, eprosartan, irbesartan, olmesartan, and telmisartan. They differ in their affinity for the AT1R (Kd from 2 nM (irbesartan) to 10 nM (losartan)), their selectivity for AT1 and AT2 receptors (from 1000:1 (losartan) to 30,000:1 valsartan)), their biological half-life (from 5h (eprosartan) to 24h (telmisartan)), and their metabolism (mostly involving CYP2C9 for losartan, candesartan, irbesartan, azilsartan, and valsartan, while olmesartan is a substrate of SLCO1B1 (which may impact its tissue distribution) [150,151]. 

## 10. Mineralocorticoid Receptor Antagonists 

Antimineralocorticoids including spironolactone and eplerenone, are potassium-sparing diuretics that block the effects of aldosterone on the mineralocorticoid receptors. Mineralocorticoid receptors are expressed in epithelial and non-epithelial tissues including kidney, salivary gland, airway epithelia, gut, brain, hypothalamus, heart, and others [62,152,153,154]. Spironolactone and eplerenone can block both epithelial and non-epithelial actions of aldosterone [155]. Compared to spironolactone, eplerenone is a next-generation aldosterone antagonist selective for the aldosterone receptor, with a shorter half-life and no active metabolite [62,155,156]. Eplerenone is mainly metabolized by CYP3A4 and is subjected to drug–drug interactions with inhibitors or strong affinity substrates of CYP3As (increased eplerenone AUC 2- to 5-fold) [157]. Spironolactone and eplerenone use can increase serum potassium, which may lead to clinically relevant hyperkaliemia [155]. In addition to standard therapy, a blockade of aldosterone by mineralocorticoid receptor antagonists has been associated with improved cardiovascular functions and survival rates among patients with severe heart failure [158,159].

## 11. Renin Antagonist 

Aliskiren is an antagonist to renin and is indicated for the treatment of hypertension in adults and children six years of age and older [160]. Aliskiren has poor bioavailability (2.5%) and most of the drug is metabolized by CYP3A4 [160]. In contrast to other antihypertensive agents and RAAS modulators, aliskiren, a direct inhibitor of renin, simultaneously reduces Ang I, Ang II, and plasma renin activity [161,162]. ACE inhibitors do not provide a full blockade of Ang II, and ARBs block the negative feedback of Ang II upon renin leading to increase in Ang II levels. Hence, both can lead to increases in plasma renin activity [160,161].

## 12. Effects of RAAS Modulators on ACE2 Expression 

Several modulators of the RAAS interact at different sites in the cascade and result in various effects on ACE2 levels (Figure 2). Table 1 summarizes major studies showing the effects of RAAS modulators on ACE2 mRNA, protein levels, and activity. Though a majority of these studies have been carried out in in vitro systems, they contribute significantly to our understanding of the role of RAAS modulators on ACE2 expression at a molecular level. Effects of these drugs on ACE2 depend on the system studied, the stage of disease progression, and the drug used. Altogether, case series and epidemiological and clinical observations of patients with SARS-CoV-2 have reported that older ages and presence of comorbidities, including cardiovascular disease, diabetes, and chronic hypertension, are associated with poor prognosis and a high rate of mortality [163]. Patients with these comorbidities are usually treated with RAAS modulators, including ACE inhibitors, ARBs, mineralocorticoid receptor antagonists, and renin antagonists (some restrictions in diabetic patients or treated with other anti-hypertensive drugs are identified in the monograph of the respective drugs). The impact of these drugs on ACE2 plasma levels or expression are still a matter of controversy, although recent studies tend to demonstrate that ACE2 plasma levels are not increased by RAAS modulators [96].

## 13. RAAS Modulators and Pneumonia Risk

A closer look at the association between the use of ACE inhibitors and the risk of pneumonia-related mortality has been the subject of several publications for more than 20 years [164]. Pleiotropic effects of ACE inhibitors on various regulatory systems could potentially be beneficial, but could also be considered potentially deleterious. For instance, silent aspiration is prevalent among elderly patients with an impaired cough reflex, a known predisposing factor for pneumonia [165,166]. It has been proposed that a cough induced by ACE inhibitors due to the block of substance P degradation could be beneficial [167,168]. Others have suggested that the anti-inflammatory properties of ACE inhibitors (decreased production of inflammatory molecules such as reactive oxygen intermediates, adhesion molecules, growth factors, chemokines, and cytokines), when used chronically, could prevent the development of lung fibrosis and improve the lung’s ability to respond to additional insults, such as infections [169,170,171,172,173]. Some researchers argue that the ARBs share several of these properties (minus the cough reflex) and could also be beneficial in the case of lung infections [174].

Answers to some of these hypotheses have been provided in several clinical studies. At the beginning of the 2000s, a series of randomized controlled trials demonstrated that patients on ACE inhibitors had a decreased incidence of pneumonia [175,176,177]. Around the same time, other studies could not confirm such protective effects [178,179,180]. For instance, the study by Liappis et al. demonstrated that ACE inhibitor use was associated with decreased rates in survival (odds ratio of 2.1; 95% confidence interval of 1.1–4.3) [181]. In a follow-up study, Mortensen et al. suggested that the reason for such divergent results could be because ACE inhibitors may not be all the same [182]. Results obtained from 186 patients hospitalized with pneumonia undergoing treatment with an ACE inhibitor demonstrated that a 30-day mortality rate (9.2%) was associated with the use of lipophilic ACE inhibitors (quinapril, fosinopril, ramipril), but not of hydrophilic ACE inhibitors (lisinopril, captopril, enalapril). 

Mortensen et al. also proposed that patients with either community- or hospital-acquired pneumonia had favorable outcomes if treated with ACE inhibitors and HMG-CoA reductase inhibitors [183]. A large study conducted by Lui et al., in 10,990 cases of patients hospitalized for pneumonia, concluded that there was no association between the use or the cumulative dose of ACE inhibitors or ARBs and risk of pneumonia [184]. Another large study conducted in 215,225 patients concluded that there was an association between the use of ACE inhibitors and ARBs and a delay in the progression of pulmonary complications in vulnerable populations [185]. Finally, a meta-analysis performed by Caldeira et al. in 2012 looked at 37 studies demonstrating that ACE inhibitors were associated with a significantly reduced risk of pneumonia in comparison to control treatment and ARBs [186]. Compared to control treatments, both ACE inhibitors (7 studies) and ARBs (1 study) were associated with a decrease in pneumonia-related mortality, without differences in intervention. They concluded that the best evidence available pointed towards a putative protective role of ACE inhibitors, but not ARBs, in risk of pneumonia. 

## 14. Other Drug Classes and ACE2 Modulation: HMG-CoA Reductase Inhibitors

As mentioned previously, Mortensen et al. demonstrated that patients with either community- or hospital-acquired pneumonia had favorable outcomes if treated with ACE inhibitors and HMG-CoA reductase inhibitors (statins) [183]. In an extensive review, Fedson presents new paradigms about the treatment of serious infection diseases, including Ebola, and how we should learn from the past [187]. He strongly advocates that use of drugs such as ACE inhibitors and statins would have a major impact on patient outcomes for those experiencing severe virus infections. In silico studies performed by Reiner et al., demonstrated that statins, especially pitavastatin, have a binding energy to SARs-CoV-2 main protease—a key coronavirus enzyme—that is even greater than that of protease or polymerase inhibitors [188]. Statins have been suggested to possess pleiotropic effects including inhibition of deleterious effects associated with RAAS overactivation. Modulation of ACE2 induced by statins is mostly described in animal experiments, cell experimental models and/or under disease situations [189,190,191,192]. Thus, the upregulation induced by statins might represent a normalization of ACE2 levels. Another pleiotropic effect of statins is the modulation of CD-147, which is another cell surface protein beside ACE2 that could interact with coronavirus entry [193,194,195,196]. In a viewpoint publication, Katsiki et al. presented elements in support of maintaining the use of statins in patients with COVID-19 due to their beneficial effects on inflammation, vascular, heart, and lung functions [197].

## 15. Getting Answers to the Central Question

Whether ACE inhibitor and/or ARB use is clearly beneficial, neutral, or deleterious in the context of COVID-19-induced pneumonia remains to be seen, and this represents a significant gap in the literature that some have described as a possible “double-edged sword” [198,199]. As mentioned previously, pharmacologic RAAS inhibition may upregulate ACE2 expression, which may amplify the virulence of SARS-CoV-2 within the pulmonary systems due to virus S-protein binding to ACE2 in order to gain entry into cells [26,30,45,130]. In this scenario, it is proposed that the use of ACE inhibitors and ARBs might predispose patients to a more severe SARS-CoV-2 infection [12]. In contrast, as explained above, mechanistic studies from related coronaviruses have shown that RAAS activation and resulting reduction in ACE2 expression plays an essential role in the pathogenesis of lung injury, acute respiratory distress syndrome, and fulminant myocarditis after SARS-CoV infection due to toxic overaccumulation of Ang II [72]. In this situation, using RAAS modulators could lead to elevated ACE2 expression and Ang-(1–7) levels, increasing their cardioprotective effects, which may be beneficial in protecting against infection.

Answer to these questions have started to be clearer as a recent communication by the World Health Organization (WHO) recognizes concerns that have been raised between the use of RAAS modulators and increased susceptibility to SARS-CoV-2 and the likelihood of severe COVID-19 illness [16]. A review of 11 observational studies (8 of which were conducted in China), led them to conclude “*that there is low-certainty evidence that patients on long-term therapy with ACE inhibitors or ARBs are not at risk of poor outcomes from COVID-19”.* Furthermore, from the results of their recent observational study in Medicare and commercially insured populations, Khera et al. conclude that *“the use of ACE inhibitors and ARBs was not associated with the risk of hospitalization or mortality among those infected with SARS-CoV-2”* [17]. In addition, they suggest that the risk of hospitalization was lower in ACE inhibitor users. The meta-analysis conducted by Mackey et al. led them to conclude that *“High-certainty evidence suggests that ACE inhibitor or ARB use is not associated with more severe COVID-19 disease, and moderate-certainty evidence suggest no association between use of these medications and positive SARS-CoV-2 test results among symptomatic patients. Whether these medications increase the risk for mild or asymptomatic disease or are beneficial in COVID-19 treatment remains uncertain”* [18].

## 16. Conclusions

Until we have clear evidence, WHO and numerous professional organizations urged patients at risk of, or currently infected with, HCoV such as COVID-19 to continue their RAAS modulator therapy as prescribed [15,16]. Based on premises presented here, there are ongoing clinical trials across the world investigating the efficacy and safety of RAAS modulators in COVID-19 prevention and treatment (Table 2; a constant update of this information could be found at https://trhc.spprdi.com/covid19/). There is limited current evidence available in patients tested positive for SARS-CoV-2 of the benefit or harm of RAAS modulators. Well-designed prospective and retrospective observational studies, in addition to randomized controlled clinical trials, will be essential for creating a guidance for use or non-use of RAAS modulators in SARS-CoV-2 infection [200]. Results from COVID-19 clinical studies may provide key insights into the differences between classes of drugs (ACE inhibitors, ARBs, mineralocorticoid receptor antagonists, statins) or the differences among drugs within the same drug class (lipophilicity, tissue vs. serum ACE inhibition) or whether outcomes from SARS-CoV-2 pneumonia in patients treated with RAAS modulators differ from other types of virus-induced pneumonia. 

In conclusion, maintaining high levels of tissue ACE2 activity appears to be beneficial for preventing exacerbated inflammatory response during COVID-19 infection, while increased plasma ACE2 levels are associated with cardiovascular complications [96]. Both sex- and racial-related genetic variants are observed in *ACE2*; some variants may also confer resistance [109].

The close relationship between the mechanism of SARS-CoV-2 entry into the cells, ACE2, and the significant number of patients affected by COVID-19 with comorbidities requiring use of RAAS modulators, presents a conundrum that can only be solved by focused clinical studies. Though the current level of evidence is not strong enough to build clinical guidelines, it provides a clear direction and roadmap for future investigations. 

**Table 1:** Clinical and experimental studies investigating impacts of several cardiopulmonary diseases and RAAS modulators on ACE2 expression and activity.

Drug Class	System Studied	Condition	Drugs	Impact on ACE2
ACE-I	In vitro in CHO cells [75]	Healthy	Captopril, Enalapril, Lisinopril	No change in ACE2 activity
	In vivo in rat renal cells [201]		Lisinopril, low sodium diet, or both	Combination lisinopril and low sodium diet decreased ACE2 mRNA
	In vivo in rat cardiac (LV) cells [202]		Lisinopril	Increase in ACE2 mRNA
	In vivo in rat cardiac cells [73]	MI	Ramipril	No effect on ACE2 mRNA or activity
	In vivo in rat cardiac (LV) and plasma cells [203]		Enalapril	Prevention of decrease in ACE2 mRNA and activity 8 weeks post-MI
	In vivo in rat pulmonary tissue and in vitro in rat PMVECs [204]	ALI	Captopril	Increase in ACE2 protein level
	In vivo in human intestinal cells [205]	Likely HTN	Any ACE-Is	Increase in intestinal ACE2 mRNA
	In vivo in human urine [206]	HTN	Enalapril	No change in ACE2 protein level in urine
	In vivo in human heart (LV) tissue [207]	HF	Any ACE-Is	ACE and ACE2 immunoreactivity were quantitatively increased in cardiac tissue from failing hearts (*n* = 5) due to ischemic heart disease compared to the non-ischemic heart controls (*n* = 3)
ARB	In vivo in rat cardiac (LV) cells [208]	Healthy	Losartan	Increase in ACE2 mRNA and activity
	In vivo in rat cardiac (LV)/ renal cells [209]	HTN		Potentiation of renal upregulation of ACE2 mRNA
	In vivo in rat aorta/carotid artery cells [210]		Olmesartan	Increase in ACE2 mRNA and activity
	In vivo in rat cardiac (aorta) cells [211]		Telmisartan	Decrease in ACE2 activity
	In vivo in rat cardiac cells	MI	Losartan, Olmesartan [212]	Increase in ACE2 mRNA
			Valsartan [213]	No effect on ACE2 mRNA or activity
		HF	Eprosartan [214]	Increase in ACE2 activity
	In vivo in rat BALF [215]	ARDS	Losartan	Increase in ACE2 activity
	In vitro in human UASMCs [211]	Healthy	Telmisartan	Decrease in ACE2 activity
	In vivo in human intestinal cells [205]	Likely HTN	Any ARBs	No changes in intestinal ACE2 mRNA
	In vivo in human urine [206]	HTN	Candesartan, Losartan Olmesartan, Telmisartan, Valsartan	ACE2 protein level was increased after treatment (>1 year) [Olmesartan only]
ACE-I + ARB	In vivo in rat cardiac (LV) cells [208]	Healthy	Lisinopril + Losartan	Increase in ACE2 activity, but decrease in ACE2 mRNA
	In vivo in rat cardiac cells [213]	MI	Ramipril + Valsartan	No effect on ACE2 mRNA or protein levels
MRA	In vivo in mice peritoneal macrophages/ cardiac cells [216]	Healthy	Eplerenone	Increase in ACE2 mRNA and activity
	In vivo in rat cardiac cells [214]	HF	Spironolactone	Trending increase in ACE2 activity (*p* = 0.067)
	In vivo in human monocyte-derived macrophages [216]			Increase in ACE2 mRNA and activity
	In vivo in human plasma [217]		Any MRAs	MRAs were found to independently associate with plasma sACE2 plasma activity
RI	In vivo in rat renal cells [218]	DN	Aliskiren	Decrease in ACE2 activity
None	In vivo in mice pulmonary cells and in vitro in Vero E6 cells [72]	SARS-CoV LRTI		SARS-CoV Spike protein binding to ACE2 in mice lungs in vivo or in cell lines resulted in reduced surface ACE2 protein levels.
	In vitro in human A549 alveolar epithelial cells [72]			SARS-CoV Spike protein binding to ACE2 in human AECs resulted in reduced surface ACE2 protein level.
	In vivo in mice cardiac cells [219]			Decrease in ACE2 mRNA and activity
	In vivo in human cardiac cells [219]			Decrease in ACE2 protein level
	In vivo in bronchoalveolar lavage fluid of rats [215]	ARDS		In bronchoalveolar lavage fluid from LPS-exposed rats, ACE activity was augmented (9-fold) while ACE2 activity was reduced (30-fold) vs controls, decreasing the ACE/ACE2 activity ratio
	In vivo in rat cardiac (LV)/ plasma cells [203]	MI		ACE2 mRNA and activity were increased at week 1 post MI compared to controls, however, they were lower than controls at week 8.
	In vivo in mice cardiac cells [220]	HF	Ang-(1–7)	Adverse remodeling in pressure-overloaded ACE2-deficient hearts is facilitated by a combination of pathological effects of Ang II on cardiac cells that can be successfully inhibited by Ang-(1–7).
	In vivo in bronchoalveolar lavage fluid of rats [215]	ARDS		In bronchoalveolar lavage fluid from LPS-exposed rats, the exposure to cAng-(1–7) increased the ACE2 activity compared to the placebo group

ACE-I: angiotensin-converting enzyme inhibitor; ALI: acute lung injury; Ang-(1–7): Angiotensin-(1–7); Ang II: angiotensin II; ARB: angiotensin-receptor blockers; ARDS: acute respiratory distress syndrome; CHO: Chinese hamster ovary; DN: diabetic nephropathy; EMC: encephalomyocarditis; HF: heart failure; HTN: hypertension; IHD: ischemic heart disease; LPS: lipopolysaccharide; LRTI: lower respiratory tract infection; LV: left ventricle; MI: myocardial infarction; MRA: mineralocorticoid receptor antagonist; mRNA: messenger RNA; NA: not applicable; PMVECs: pulmonary microvascular endothelial cells; RAAS: renin angiotensin-aldosterone system; rhACE2: recombinant human angiotensin-converting enzyme 2; RI: renin inhibitor; RSV: respiratory syncytial virus; SARS-CoV: severe acute respiratory syndrome coronavirus; UASMCs: umbilical artery smooth muscle cells. The yellow and blue colors represent experiments conducted in animals or human, respectively.

**Table 2:** Current or planned registered clinical trials in COVID-19 prevention and treatment investigating efficacy and/or safety of RAAS modulators.

Drug Class	Treatment	Type of Study	COVID-19 Status	Setting	Severity	Phase	Country	Trial Status (Expected N)	Trial ID
ACE-I	Ramipril vs. placebo	Triple-blind, placebo-controlled; efficacy	Confirmed	HOSP or ED	Non-severe	2	US	Not yet recruiting (560)	NCT04366050
	Captopril vs. SOC	Open label; efficacy	Confirmed	HOSP	ARDS	2	France	Not yet recruiting (230)	NCT04355429
	Captopril or enalapril vs. CQ	Open label; efficacy	Confirmed	Unspecified	Unspecified	3	Egypt	Not yet recruiting (60)	NCT04345406
ARB	Valsartan vs. placebo	Quadruple-blind, placebo-controlled; efficacy	Confirmed	HOSP	Mixed	4	Netherlands	Recruiting (651)	NCT04335786
	Chloroquine/Hydroxychloroquine vs. LPV/r vs. SOC vs. Rivaroxaban vs. TP vs. Candesartan vs. non-RAS AHT vs. Clazakizumab vs. Placebo	Open label; active-controlled; efficacy	Confirmed	Healthy, Outpatient, HOSP	Mixed	2/3	Austria	Recruiting (500)	NCT04351724
	Telmisartan vs. Placebo	Triple-blind, placebo-controlled; efficacy and safety	Confirmed	Outpatient	Non-severe	2	US	Not yet recruiting (40)	NCT04360551
	SOC vs Telmisartan + SOC	Open label; efficacy	Confirmed	Unspecified	Unspecified	2	Argentina	Recruiting (400)	NCT04355936
	Hydroxychloroquine vs. Azithromycin vs. Telmisartan vs. SOC	Open label; efficacy	Confirmed	HOSP	Mixed	3	France	Not yet recruiting (1600)	NCT04359953
	Vitamins vs. Hydroxychloroquine vs. Imatinib vs. Favipiravir vs. Telmisartan	Open label, multi-stage, superiority; efficacy and safety	Confirmed	Outpatient	Mild	3	France	Recruiting (1057)	NCT04356495
	Losartan	Open label; safety	Confirmed	ICU or HOSP	ARDS	1	US	Recruiting (50)	NCT04335123
	Losartan vs. Placebo	Quadruple-blind, placebo-controlled; efficacy	Confirmed	Outpatient	Non-severe	2	US	Recruiting (580)	NCT04311177
	Losartan vs. Placebo	Quadruple-blind, placebo- controlled; efficacy	Confirmed or Suspected	HOSP	ARDS	2	US	Recruiting (200)	NCT04312009
	SOC vs. Losartan + SOC	Open label; efficacy	Confirmed	HOSP	Mild/ Moderate	4	US	Recruiting (200)	NCT04340557
	Losartan vs. Amlodipine	Open label; efficacy and safety	Confirmed	HOSP	Non-severe	3	Iran	Recruiting (100)	IRCT201808020 40678N4
	SOC vs. ASA vs. Losartan vs. Simvastatin vs. ASA + losartan vs. ASA + Simvastatin vs. Losartan + Simvastatin vs. ASA + Losartan + Simvastatin	Open label, factorial; efficacy	Confirmed or Suspected	HOSP	Mixed	3	Nigeria, Pakistan	Not yet recruiting (10,000)	NCT04343001
	SOC + LPV/r vs. SOC + Hydroxychloroquine vs. SOC + Losartan vs. SOC + Placebo	Quadruple-blind, placebo-controlled; efficacy	Confirmed	HOSP	Mixed	2/3	US	Recruiting (4000)	NCT04328012
	Hydroxychloroquine + Azithromycin vs. Hydroxychloroquine + Doxycycline vs. Hydroxychloroquine + Clindamycin vs. Hydroxychloroquine + Clindamycin + Primaquine low dose vs Hydroxychloroquine + Clindamycin+ Primaquine high dose vs. Remdesivir vs. Tocilizumab vs. Methylprednisolone vs. Interferon-α2B vs. Losartan vs. Plasma	Single-blind, factorial; efficacy	Confirmed	HOSP	Unspecified	2/3	US	Enrolling by invitation (500)	NCT04349410
	ARBs vs. SOC	Single-blinded; parallel assignment; safety and efficacy	Confirmed	HOSP	Unspecified	4	Australia	Recruiting (605)	NCT04394117
	ARBs vs. SOC	Prospective observational; safety, efficacy, ACE2 activity	Confirmed	HOSP	COVID+ ARDS	NA	France	Recruiting (100)	NCT04337190
	Chloroquine + Losartan vs. Chloroquine	Randomized, double blinded; safety and efficacy	Confirmed	HOSP	Unspecified	2	Mexico	Recruiting (20)	NCT04428268
ACE-I or ARB	D/C ACE-I/ARB and switch to CCB or TZD vs continue ACE-I/ARB	Open label; prevention	COVID-19 naive	Healthy	NA	4	Ireland	Recruiting (2414)	NCT04330300
	D/C vs continue ACE-I/ARB	Single-blind; efficacy	Confirmed or Suspected	HOSP	Mixed	NA	US	Enrolling by invitation (152)	NCT04338009
	D/C vs continue ACE-I/ARB	Single-blind; efficacy	Confirmed	HOSP	Mild	4	Austria, Germany	Recruiting (208)	NCT04353596
	D/C vs continue ACE-I/ARB	Single-blind; efficacy	Confirmed	HOSP	Mixed	NA	Denmark	Recruiting (215)	NCT04351581
	D/C vs continue ACE-I/ARB	Open label; efficacy	Confirmed	HOSP	Mild	NA	Brazil	Recruiting (500)	NCT04364893
	D/C vs continue ACE-I/ARB	Open label; efficacy and safety	Confirmed	HOSP	Mild	3	France	Recruiting (554)	NCT04329195
	Use vs no use of ACE-I/ARB	Prospective observational; prognosis	Confirmed	HOSP	Unspecified	NA	Saudi Arabia	Recruiting (226)	NCT04357535
	ACE-I/ARB/ direct renin inhibitors (DRi)	Prospective observational; prognosis and efficacy (hypertension)	Confirmed	Private practice	Unspecified	NA	Ukraine	Recruiting (10)	NCT04364984
	ARB/ACE-I vs anti-malarial drugs	Prospective, observational; prognosis	Confirmed	HOSP	Unspecified	NA	France	Not yet recruiting (6,000,000)	NCT04356417
	ARB/ ACE-I/ influenza vaccine vs. SOC	Prospective, observational; efficacy	Confirmed	HOSP	Unspecified	NA	Spain	Recruiting (2547)	NCT04367883
	ACE-I/ARB +SOC vs. SOC	Retrospective, case-control; severity and mortality of/ from COVID	Confirmed	Unspecified	ARDS vs. non-ARDS COVID	NA	Italy	Not yet recruiting (5000)	NCT04318418
	ACE-I/ARB +SOC vs. SOC	Retrospective, case-control; safety	Confirmed	HOSP	Unspecified	NA	France	Not yet recruiting (700)	NCT04374695
	ACE-I/ARB + SOC vs. SOC	Open-label, case control; prognosis	Confirmed	HOSP	Any severity	NA	Italy	Recruiting (2000)	NCT04331574
MRA	Spironolactone vs. Placebo	Triple-blind, placebo-controlled; efficacy	Confirmed	ICU	ARDS	4	Turkey	Not yet recruiting (60)	NCT04345887
	Bromohexine + spironolactone vs. standard therapy	Single-blind, parallel assignment; efficacy	Confirmed	HOSP	Mild to moderate	3	Russia	Recruiting	NCT04424134
MAS receptor agonist	Angiotensin-(1–7) vs Placebo	Triple-blind, placebo-controlled; efficacy and safety	Confirmed or Suspected	ICU	ARDS	2/3	Belgium	Not yet recruiting (60)	NCT04332666
	Plasma derived Angiotensin-(1–7) vs. SOC	Open label; efficacy	Confirmed	HOSP	NA	NA	Turkey	Recruiting	NCT04375124
Recombinant ACE-2	Recombinant human ACE-2 (APN01) vs. placebo	Double-blind, placebo-controlled; efficacy	Confirmed	HOSP	Mixed	2	Austria, Denmark, Germany	Recruiting (200)	NCT04335136

Key: ACE-I: angiotensin-converting enzyme inhibitor; AHT: antihypertensive; ARB: angiotensin II receptor blocker; ARDS: acute respiratory distress syndrome; ASA: acetylsalicylic acid; BCC: calcium channel blockers; D/C: discontinuation; ED: emergency department; HOSP: hospitalized; HTN: hypertension; LPV/r: lopinavir/ritonavir; ICU: intensive care unit; MRA: mineralocorticoid receptor antagonist; NA: not applicable; RAS: renin angiotensin system; SOC: standard of care; TP: thromboprophylaxis; TZD: thiazolidinedione. NCTXXXXXXXX refers to registry number at the NIH ClinicalTrials.gov website. IRCT201808020 40678N4 refers to registry number at the Iranian Registry of Clinical Trials. An update of the information listed in this table can be found at https://trhc.spprdi.com/covid19/. * Excluded the following types of studies: retrospective case-control, prospective cohort with previous ARB use, and studies in which RAAS modulator use was not a primary focus. Table was updated on 6/23/20.
